# Development to Term of Cloned Cattle Derived from Donor Cells Treated with Valproic Acid

**DOI:** 10.1371/journal.pone.0101022

**Published:** 2014-06-24

**Authors:** Juliano Rodrigues Sangalli, Marcos Roberto Chiaratti, Tiago Henrique Camara De Bem, Reno Roldi de Araújo, Fabiana Fernandes Bressan, Rafael Vilar Sampaio, Felipe Perecin, Lawrence Charles Smith, Willian Allan King, Flávio Vieira Meirelles

**Affiliations:** 1 Departamento de Medicina Veterinária, Faculdade de Zootecnia e Engenharia de Alimentos, Universidade de São Paulo, Pirassununga, São Paulo, Brazil; 2 Departamento de Cirurgia, Faculdade de Medicina Veterinária e Zootecnia, Universidade de São Paulo, São Paulo, São Paulo, Brazil; 3 Department of Biomedical Science, Ontario Veterinary College, University of Guelph, Ontario, Canada; 4 Departamento de Genética e Evolução, Centro de Ciências Biológicas e da Saúde, Universidade Federal de São Carlos, São Carlos, Brazil; 5 Departamento de Genética, Faculdade de Medicina de Ribeirão Preto, Universidade de São Paulo, Ribeirão Preto, São Paulo, Brazil; 6 Centre de recherche em reproduction animale, Faculté de médecine vétérinaire, Université de Montréal, St. Hyacinthe, Québec, Canada; Faculty of Animal Sciences and Food Engineering, University of São Paulo, Pirassununga, SP, Brazil, Brazil

## Abstract

Cloning of mammals by somatic cell nuclear transfer (SCNT) is still plagued by low efficiency. The epigenetic modifications established during cellular differentiation are a major factor determining this low efficiency as they act as epigenetic barriers restricting reprogramming of somatic nuclei. In this regard, most factors that promote chromatin decondensation, including histone deacetylase inhibitors (HDACis), have been found to increase nuclear reprogramming efficiency, making their use common to improve SCNT rates. Herein we used valproic acid (VPA) in SCNT to test whether the treatment of nuclear donor cells with this HDACi improves pre- and post-implantation development of cloned cattle. We found that the treatment of fibroblasts with VPA increased histone acetylation without affecting DNA methylation. Moreover, the treatment with VPA resulted in increased expression of *IGF2R* and *PPARGC1A*, but not of *POU5F1*. However, when treated cells were used as nuclear donors no difference of histone acetylation was found after oocyte reconstruction compared to the use of untreated cells. Moreover, shortly after artificial activation the histone acetylation levels were decreased in the embryos produced with VPA-treated cells. With respect to developmental rates, the use of treated cells as donors resulted in no difference during pre- and post-implantation development. In total, five clones developed to term; three produced with untreated cells and two with VPA-treated cells. Among the calves from treated group, one stillborn calf was delivered at day 270 of gestation whereas the other one was delivered at term but died shortly after birth. Among the calves from the control group, one died seven days after birth whereas the other two are still alive and healthy. Altogether, these results show that in spite of the alterations in fibroblasts resulting from the treatment with VPA, their use as donor cells in SCNT did not improve pre- and post-implantation development of cloned cattle.

## Introduction

In 1997, cloning of mammals by somatic cell nuclear transfer (SCNT) was shown feasible, becoming a promising technology because of its applications in medicine (e.g. derivation of patient-specific embryonic stem cells) and transgenesis [1]. However, SCNT is still plagued by low efficiency, as a multitude of successful steps are needed for a cloned animal to be born healthy [2]. These include oocyte-mediated reprogramming, pregnancy establishment, development of a functional placenta with adequate maternal–fetal interaction, finally successful delivery and adaptation to the extra uterine life [3]. Problems such as persistence of somatic cell methylation [4] and acetylation patterns in SCNT embryos [5], aberrant expression of imprinted genes [6], failure to produce a functional placenta and consequently poor fetal nutrition are frequently observed in animal clones [7]. These observations suggest that the nuclear reprogramming process is incomplete in most SCNT embryos representing an important target for strategies aiming to improve SCNT efficiency [8].

There are several epigenetic modifications that accompany cell differentiation, including DNA methylation, histone modifications (e.g. H3K9me2/3 methylation; deacetylation), incorporation of histone variants (e.g. macroH2A) and chromatin compaction. All these modifications act as epigenetic barriers restricting reprogramming of somatic nuclei [9]. Since histone deacetylation, followed by DNA compaction, is commonly associated with gene repression in differentiated cells, decondensation of chromatin is required to enable access of transcriptional regulators to genomic targets essential for successful reprogramming of somatic nuclei [10], [11]. Corroborating this hypothesis, most factors that promote chromatin decondensation, including histone deacetylase inhibitors (HDACis), have been found to increase nuclear reprogramming efficiency, making their use common to improve SCNT rates [12], [13].

The use of HDACis, including Valproic Acid (VPA) [14], Sodium Butyrate (NaBu) [15], Trichostatin A (TSA) [16] and Scriptaid [17] is associated with increased rates of successful nuclear reprogramming. An increase of up to five fold in the success rate of mouse cloning was reported when HDACi was used in SCNT [13]. For example, treatment of donor cells with TSA, markedly improved in vitro development of SCNT embryos in rabbits [18] and cattle [19]. In addition, the use of NaBu in cattle improved SCNT [15] whereas the use of VPA enhanced gene reactivation and reprogramming efficiency in pig SCNT [20] and mouse induced pluripotent stem cells (iPSCs) experiments [21]. VPA, a short-chain fatty acid, is widely used in humans as an anticonvulsant and mood stabilizer [22]. Recently, VPA was reported to be a powerful HDACi with low toxicity to the cells, both in vitro and in vivo [23]. VPA relieves HDAC-dependent transcriptional repression and causes hyperacetylation of histones in cultured cells and in vivo [24], [25]. Since histone hyperacetylation promotes neutralization of positive charges of lysine residues, this reduces their DNA binding and may contribute directly to chromatin opening and indirectly to transcriptional upregulation [26]. For this reason, VPA has been used in cattle to investigate its effect on nuclear reprogramming. Treatment of cloned embryos with VPA greatly improved blastocyst formation rate, the number of cells in the inner cell mass (ICM) and trophectoderm (TE), and cell survival in cattle [27]. Recently, Selokar et al. demonstrated in cattle that the use of donor cells treated with VPA was beneficial for efficient reprogramming using handmade cloning procedures [28]. Nonetheless, the use VPA has been limited to experiments involving pre-implantation development as end-points, with no study having evaluated its effect on development of cattle clones to term.

Herein VPA was used in SCNT to test whether the treatment of nuclear donor cells with this HDACi affects pre- and post-implantation development of cattle clones. We hypothesized that the treatment of donor cells with VPA might relax the chromatin structure turning them more amenable cells for reprogramming. As a result, based on previous studies and the low- toxicity of VPA, an increase of the global levels of histone acetylation favoring nuclear reprogramming towards higher developmental rates was expected.

## Materials and Methods

All chemicals and reagents used were purchased from Sigma-Aldrich Chemical Company (St. Louis, MO, USA) unless otherwise stated. In vitro experimental procedures were carried out in humidified incubators maintained at 38.5°C in air with 5% CO_2_. Each experiment consisted of at least three replicates. The experiments were conducted in accordance with the International Guiding Principles for Biomedical Research Involving Animals (Society for the Study of Reproduction).

### Ethics Statement

The present study was approved by the “Ethic Committee in the use of animals” of the School of Veterinary Medicine and Animal Science of University of São Paulo, protocol number 2546/2012, which complies with the ethical principles in animal research. We adopted the International Guiding Principles for Biomedical Research Involving Animals (Society for the Study of Reproduction) as well.

### Treatment of nuclear donor cells with VPA

Nuclear donor cells were derived from a 14-year old Gir (*Bos indicus*) bull as described previously [29]. Fibroblasts at third passage were plated at a density of 7×10^4^ cells per 35-mm Petri dish in Alpha Minimum Essential Medium (α-MEM; GIBCO BRL, Grand Island, NY, USA) supplemented with 10% (v/v) fetal calf serum (FCS) and 50 µg/ml gentamicin sulfate. After 48 h post plating, the medium was changed to α-MEM supplemented with 0.5% FCS and 50 µg/ml gentamicin sulfate, and the cells were cultured for another three days to arrest the cell cycle at the G1/G0 stages. The treatment with VPA was performed by adding 0, 1, 2 or 5 mM of VPA to the culture medium 24 h before the end of the culture. Based on the results from an MTT analysis (see below), only 0 mM (control) and 2 mM (treated) of VPA were used in the following experiments.

### MTT cell proliferation assay

To evaluate fibroblast viability and proliferation after VPA treatment, we used the 3-(4,5-dimethylthiazol-2-yl)-2, 4-diphenyl-tetrazolium bromide assay [30] (MTT) as previously described [29], with a few modifications. Briefly, fibroblasts were seeded in 96-well plates at 7×10^3^ cells per well and treated with 0, 1, 2 and 5 mM of VPA as described above. Thereafter, 100 µl of the MTT solution (0.5 mg/ml final concentration) were added into each well of the 96-well plates and cultured in a humidified incubator with 5% CO_2_ at 38.5°C. After 3 h, the MTT solution was removed and 200 µl of acidic isopropanol (0.04 M HCl in absolute isopropanol) were added into each well to dissolve the formed formazan crystals. Each plate was read using a spectrophotometer Thermo Scientific Multiskan FC (Thermo Scientific, Wilmington, DE, USA) at 570 nm wavelength. Absorbance values are expressed as percentages in relation to a control group (without VPA).

### Somatic cell nuclear transfer

Somatic cell nuclear transfer was performed as described by Sangalli et al. [29]. Briefly, cumulus-oocyte complexes (COCs) were aspirated from ovaries obtained from local abattoirs and selected based on morphological characteristics. COCs were then subjected to in vitro maturation (IVM) and the oocytes denuded of cumulus cells by gentle pipetting. The oocytes with visible first polar body (PB) were enucleated by removing the 1^st^ PB and metaphase-II (MII) plate by gentle aspiration using a 15-µm (internal diameter) glass pipette (ES transferTip; Eppendorf, Hamburg, Germany). Enucleated oocytes were reconstructed by injection of a single fibroblast (previously untreated or treated with VPA) into the perivitelline space. The resulting couplet was fused in electrofusion solution (0.28 M mannitol, 0.1 mM MgSO_4_, 0.5 mM HEPES and 0.05% BSA in ultra-pure water) by applying one pulse of alternating current (0.05 kV/cm for 5 s) and two pulses of continuous current (1.75 kV/cm for 45 µs). Successfully fused couplets were fixed immediately before activation (see below) or artificially activated 26 h post-IVM. Artificial activation was performed by treatment with 5 µM ionomycin for 5 min, washing in 3% BSA solution and treatment with 2 mM of 6-dimethylaminopurine (6-DMAP) for 3 h [29]. Presumptive zygotes were cultured for an additional period of 2 h post-activation (h.p.a.) and fixed for immunocytochemistry analysis (5 h.p.a.) or cultured for 7 days in SOF supplemented with 2.5% v/v FCS, 0.5% bovine serum albumin (BSA), 0.2 mM sodium pyruvate and 50 µg/ml gentamicin sulfate. Cleavage and blastocyst rates were evaluated at 48 and 168 h.p.a., respectively. In all experiments, parallel parthenogenetic embryos were generated and used as controls.

### Embryo transfer and pregnancy evaluation

Blastocysts at day 7 of development were individually transferred non-surgically into the uterus of previously synchronized recipient cows [31]. Recipients were evaluated for pregnancy by transrectal palpation/ultrasonography at 30, 60, 90 and 270 days after embryo transfer. Abortion rate was evaluated on a monthly basis.

### Immunodetection of H3K9ac and 5-methylcytosine

Immunocytochemistry for histone 3 lysine 9 acetylation (H3K9ac) was performed using fused couplets (immediately before the activation) or presumptive zygotes (5 h.p.a.). Nuclear donor cells were also evaluated by immunocytochemistry for H3K9ac and global levels of DNA methylation (5-mC) as previously reported [32]–[34], with a few modifications. Briefly, samples were fixed for 12 min in 4% (w/v) paraformaldehyde diluted in phosphate buffer solution (PBS) with 0.1% (w/v) polyvinylpyrrolidone (PBS-PVP) and permeabilized for 20 min in PBS-PVP with 1% (v/v) Triton X-100, washed in PBS-PVP containing 0.1% (v/v) Tween 20 (TW-PBS) and incubated for 15 min in 4 N HCl. Afterwards, samples were extensively washed in TW-PBS and non-specific binding sites were blocked by incubation overnight at 4°C in a blocking buffer consisting of PBS-PVP containing 0.4% (w/v) BSA. Samples were incubated for 3 h at 37°C with primary antibodies diluted at 1∶1000. DNA methylation staining (fibroblasts) was performed using the anti-mouse 5-mC (Santa Cruz Biotechnology, Santa Cruz, CA, USA) whereas the anti-rabbit H3K9ac (Millipore, Bedford, MA, USA) was used for histone acetylation staining (fibroblasts, fused couplets and presumptive zygotes). Next, samples were washed in TW-PBS and incubated for 1 h at room temperature with secondary antibodies diluted at 1∶500. The goat anti-rabbit conjugated with fluorescein isothiocyanate-FITC (Vector Laboratories, Burlingame, CA, USA) was used for histone acetylation staining and the rabbit anti-mouse conjugated with rhodamine (Santa Cruz Biotechnology) for DNA methylation staining. After several washes in TW-PBS, fibroblasts, fused couplets and presumptive zygotes were mounted on glass slides with coverslips using Prolong Antifade reagent (Invitrogen). An epifluorescent microscope (Axioplan; Carl Zeiss, Zeppelingstrasse, Germany) was used for evaluation of both DNA methylation and histone acetylation. As negative control, PBS was used in place of primary antibody.

Images were captured using a digital camera (Zeiss MC 80 DX; Carl Zeiss) and the same settings for all samples subject to the same staining (e.g. methylation or acetylation) procedure. The acetylation status was analyzed using at least 15 images of individual couplets for each experimental group. With regards to donor cells, 5 images of a monolayer of fibroblasts were evaluated for each experimental group to evaluate both acetylation and methylation status. Individual nuclei were outlined (excluding overlapping and folded nuclei) using the Adobe Photoshop v. 7 (Adobe Systems, San Jose, CA, USA). About 30 nuclei were outlined in each fibroblast image. The background was subtracted from pixel intensity in all analysis and the averaged signal intensity was calculated using the ImageJ software (ImageJ, National Institute of Health, Bethesda, MD, USA). Here, intensity refers to the degree of brightness of colored pixels (in a gray scale: 0–255; 0 = black and 255 = white) where brighter intensity indicates greater immunocytochemistry reactivity.

### Gene expression analysis

RNA was extracted from control and treated fibroblasts using TRIzol reagent (Invitrogen, Carlsbad, CA, USA) according to the manufacturer's recommendations, with a few modifications. In brief, a mix containing 100 µl of TRIzol reagent, 5 µg of linear acrylamide (Ambion Inc., Austin, TX, USA) and 5 µl of diethylene pyrocarbonate-treated H_2_O was added to each sample. The extracted RNA was directly dissolved in 10 µl of DNase I solution (Invitrogen) plus 1 unit/µl RNase OUT for DNA degradation, as suggested by the manufacturer. Then, the RNA was immediately reverse transcribed into cDNA using the High-Capacity cDNA Reverse Transcription kit (Applied Biosystems) according to manufacturer's protocol and stored at −20°C until use.

The target genes of interest were *POU5F1*, *IGF2R* and *PPARGC1A* whereas *ACTB* was used as a reference gene. Primers used for real-time reverse transcription PCR (RT-PCR) were designed using the Primer Express software v.3.1 (Applied Biosystems) based upon sequences available in GenBank (Table 1). Relative quantification of gene-specific mRNA transcripts was performed in 20-µl reactions containing 0.2 µM of primers (*POU5F1*, *IGF2R*, *PPARGC1A* and *ACTB*) plus 1× Power SYBR Green PCR Master Mix (Applied Biosystems) and 2 µl of a 8-fold diluted cDNA. All gene-specific cDNAs amplified for a particular sample were run in triplicate in the same PCR plate. A non-template control was always run in parallel with samples using 2 µl of water instead of cDNA. The following cycling conditions were applied for amplification: initial denaturation at 95°C for 10 min followed by 40 cycles consisting of 95°C for 15 sec and 60°C for 1 min. The SYBR Green fluorescence was read at the end of each extension step (60°C). Pilot experiments using five different concentrations of cDNA (spanning a 60-fold range) were run to set up real time RT-PCR conditions. The specificity of PCR products was confirmed by analysis of melting curves. Target transcript amounts were determined using the following formula: E_(target)_^−ΔCt(target)^/E_(ref)_^−ΔCt(ref)^, in which E corresponds to the amplification efficiency and ΔCt to the difference of cycle threshold (Ct) between control and treated samples. Values of Ct were averaged from sample duplicates whereas E referred to the mean efficiency estimated for each primer set (which varied from 93.6% to 95.2%) using LinRegPCR program [35]–[37]. Gene expression data from seven biological replicates are presented.

**Table 1:** Primer sequences used in real time RT-PCR.

Gene Symbol	Gene Name	Accession number	Primer Sequences (5′-3′)	Product (bp)	Annealing Temp. (°C)
*POU5F1*	POU class 5 homeobox 1	NM_174580.2	F: CAGGCCCGAAAGAGAAAGC	78	60
			R: CGGGCACTGCAGGAACA		
*IGF2R*	Insulin-like growth factor 2 receptor	NM_174352.2	F: CAGGTCTTGCAACTGGTGTATGA	83	60
			R: ACGAAGCTGATGACGCTCTTG		
*PPARGC1A*	Peroxisome proliferator-activated receptor gamma, coactivator 1 alpha	NM_177945.3	F: ACCATGCAAACCATAATCACAGGAT	82	60
			R: CTCTTCGCTTTATTGCTCCATGAAT		
*ACTB*	Actin beta	NM_173979.3	F: CAGCAGATGTGGATCAGCAAGC	91	60
			R: AACGCAGCTAACAGTCCGCC		

### Statistical analysis

All experiments were repeated at least three times. Statistical analysis was performed using the SAS System v. 9.3 (SAS/STAT, SAS Institute Inc., Cary, NC). Developmental rates were analyzed using chi-square test. Remaining data were tested for normality of residuals and homogeneity of variances, and analysed as follows. MTT and immunocytochemistry data were analysed by regression analysis and t-Student's test, respectively. Gene expression data were analyzed by one-way ANOVA followed by Tukey post-hoc test. Gene expression data are presented in natural log (Ln) scale because of the log normal distribution considered for analysis. Differences with probabilities (P)<0.05 were considered significant. In the text, values are presented as means ± the standard error of the mean (SEM).

## Results

### Valproic Acid increases proliferation/viability of donor cells

**Figure 1:**
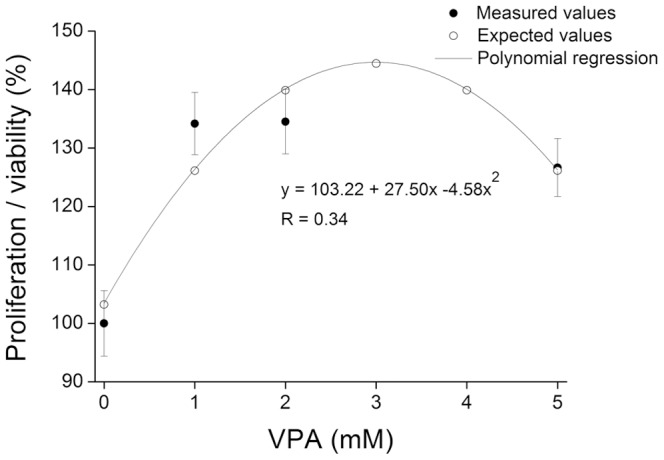
Valproic Acid increases proliferation/viability of donor cells. Proliferation/viability rates of donor cells treated with 0, 1, 2 and 5 mM VPA measured by the MTT assay. Values fitted a second grade polynomial regression (P = 0.0001).

In order to ascertain whether VPA-treated cells were suitable as nuclear donors and monitor the changes caused by VPA treatment, bovine fibroblasts were evaluated by the MTT assay after the treatment with 0, 1, 2 and 5 mM VPA for 24 h. The rate of proliferation/viability of these cells fitted (P = 0.0001) a second grade polynomial regression (Figure 1). The cells treated with 1, 2 and 5 mM VPA increased proliferation/viability by 134±5,32%, 135±5,53% and 127±4,97%, respectively, compared to untreated cells (100±5,60%). Thus it was concluded that since the treatment did not impair cell viability the cells treated with VPA were suitable as nuclear donors.

### VPA treatment increases H3K9 acetylation without affecting DNA methylation of donor cells

**Figure 2:**
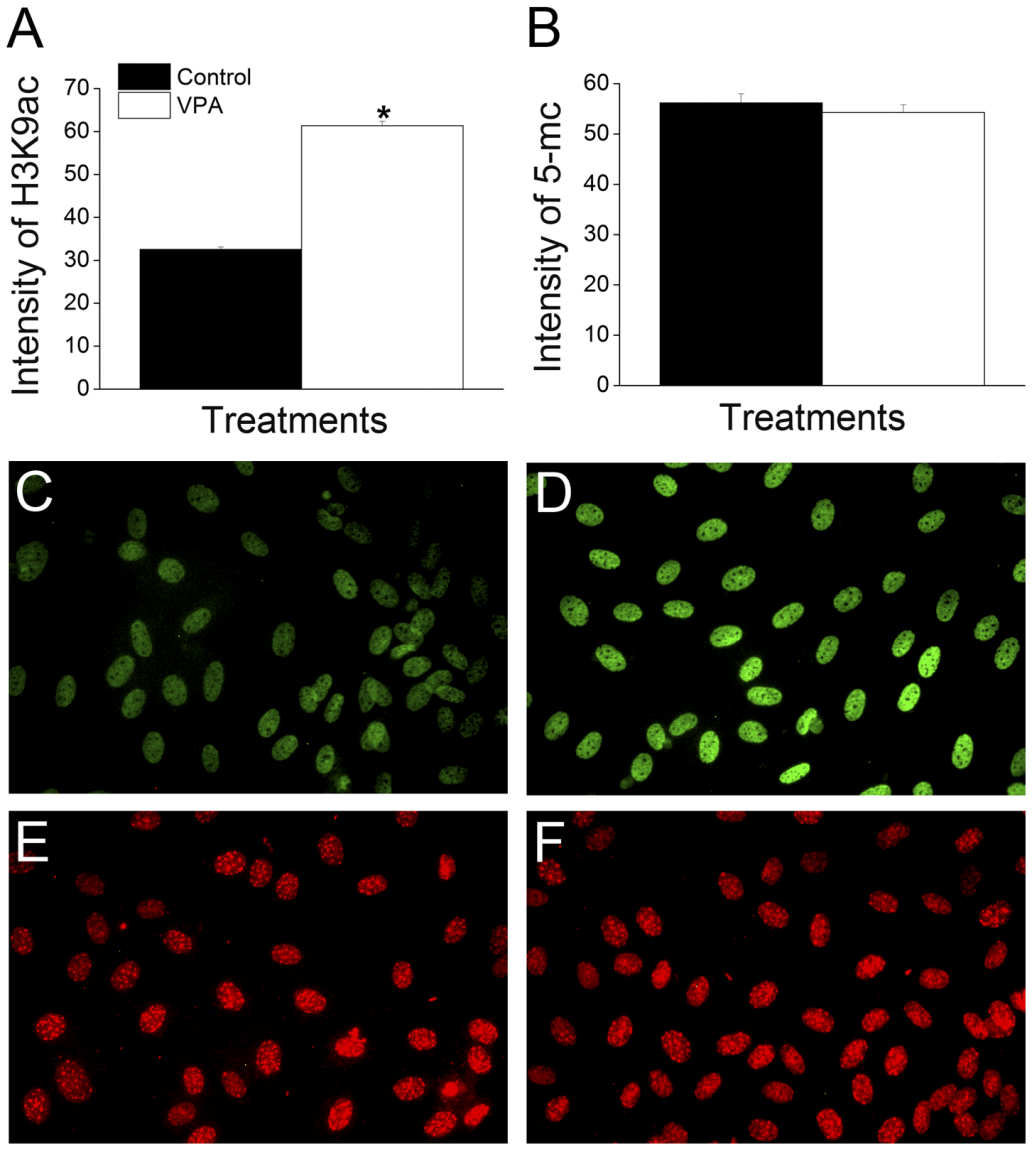
VPA treatment increases H3K9 acetylation without affecting DNA methylation of donor cells. (A) Relative average intensity of H3K9 acetylation (H3K9ac) of control and VPA-treated cells. (B) Relative average intensity of 5-methylcytosine (5-mC) of control and VPA-treated cells. (C–D) Immunofluorescence labeling for H3K9ac of control (C) and VPA-treated cells (D). (E–F) Immunofluorescence labeling for 5-mC of control (E) and VPA-treated cells (F). The (*) denotes a significant difference between experimental groups (P<0.0001). All images were taken in the same magnification (200×).

In order to confirm the effect of VPA treatment on histone acetylation, global histone acetylation levels of fibroblasts treated with 2 mM VPA were evaluated by immunocytochemistry (Figure 2). Compared to control cells, the treatment with VPA increased H3K9ac by about 1.9 fold (P<0.0001). Moreover, since VPA has been shown to decrease DNA methylation in cultured cells [38], we evaluated the global DNA methylation levels, but no differences were found between control and treated cells (Figure 2).

### Expression of *IGF2R* and *PPARGC1A* is increased in donor cells treated with VPA

**Figure 3:**
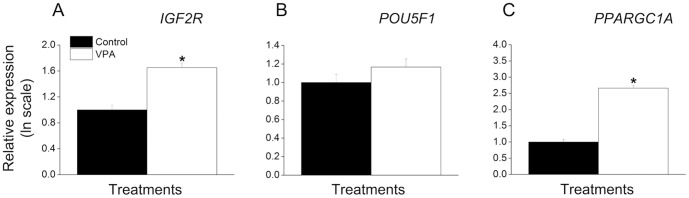
Expression of *IGF2R* and *PPARGC1A* is increased in donor cells treated with VPA. The amounts of *IGF2R* (A), *POU5F1* (B) and *PPARGC1A* (C) transcripts are expressed in relation to the control group. The asterisk (*) denotes difference between control and VPA-treated groups (P<0.0001).

In order to further understand the effect of VPA on the cells, we evaluated expression of *POU5F1*, *IGF2R* and *PPARGC1A* by real-time RT-PCR (Figure 3). The genes evaluated are known for their role in regulating fetal growth (*IGF2R*) [39], promoting pluripotency *(POU5F1)* [40] and metabolism regulation (*PPARGC1A*) [41]. With respect to *IGF2R*, it was found that the treatment with VPA increased (P<0.0001) transcript abundance by about two fold compared to control cells (Figure 3a). No difference in expression of *POU5F1* was found (Figure 3b). Finally, expression of *PPARGC1A* was increased (P<0.0001) over five fold in the cells treated with VPA (Figure 3c). In summary, treatment of cells with VPA increased expression of *IGF2R* and *PPARGC1A*, but not of *POU5F1*.

### The higher levels of histone acetylation in donor cells are not maintained after nuclear transfer

**Figure 4:**
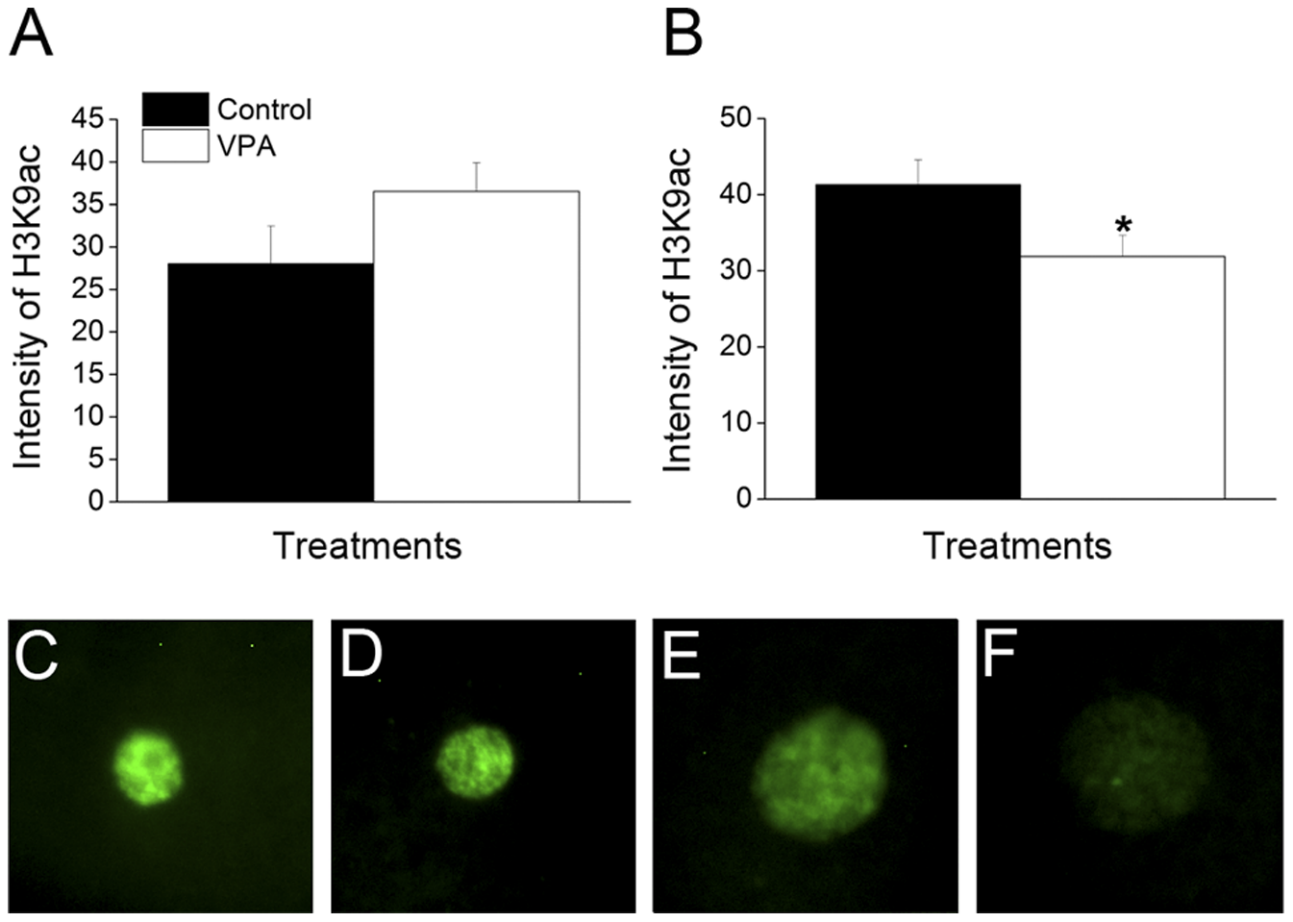
The higher levels of histone acetylation in donor cells are not maintained after nuclear transfer. (A) Relative average intensity of H3K9ac of fused couplets from control and VPA-treated groups. (B) Relative average intensity of H3K9ac of presumptive zygotes 5 h.p.a from control and VPA-treated groups. (C–D) Immunofluorescence labelling for H3K9ac of fused couplets from (C) control and (D) VPA-treated groups. (E–F) Immunofluorescence labelling for H3K9ac of presumptive zygotes 5 h.p.a. from (E) control and (F) VPA-treated groups. The asterisk (*) denotes difference between control and VPA-treated groups (P = 0.03). All images were taken in the same magnification (200×).

Fibroblasts with increased levels of histone acetylation after VPA treatment were used as SCNT donors cells. To confirm whether the higher acetylation levels were maintained after nuclear transfer, global histone acetylation levels were measured in fused couplets before and after artificial activation concerning their global histone acetylation levels (Figure 4). Although H3K9ac was marginally superior in the VPA group (28.0±4.44 versus 36.5±3.37, respectively for control and VPA), no significant difference was found between groups before activation (P = 0.12). Unexpectedly, when H3K9ac was evaluated after artificial activation (5 h.p.a.), a significant decrease of about 1.3 fold was verified in the VPA group (P = 0.03). Taken together, it appears that the higher levels of histone acetylation of donor cells treated with VPA are not maintained after nuclear transfer.

### Higher levels of histone acetylation in donor cells has no effect on pre- and post-implantation development of clones

Since the cells treated with VPA showed higher levels of histone acetylation, they were used as donors in SCNT to evaluate the effect of donor cell acetylation levels on developmental rates (Table 2). A total of 254 cloned embryos were produced in four experimental replicates, 180 controls and 168 treated, but no difference in overall rates of fusion, cleavage or blastocyst rates were found between groups. Although this finding indicates that the treatment of donor cells with VPA does not affect the development of SCNT embryos, blastocysts produced from both groups were transferred to recipients to evaluate whether the treatment affected post-implantation development (Table 3). Sixty-four blastocysts, 34 controls and 30 treated, were transferred individually into synchronized recipients. Although fewer full term gestations were obtained from VPA-treated donor cells, no significant difference was found between control and treated groups at any stage during pregnancy, further indicating that the treatment of donor cells with VPA does not affect developmental rates of cattle clones.

**Table 2:** Effect of treating nuclear-donor cells with Valproic Acid on pre-implantation developmental rates of cloned embryos.

Groups	Oocytes N	Fused embryos N (%)	Cleavage N (%)	Blastocysts N (%)
Control	180	139 (77.22)	112 (80.57)	44 (31.65)
VPA	168	115 (68.45)	90 (78.26)	38 (33.04)

Values within the same column did not differ statistically.

Fusion rate: number of embryos fused/number embryos reconstructed.

Cleavage rate: number of cleaved embryos/number of fused embryos.

Blastocyst rate: number of blastocysts/number of fused embryos.

**Table 3:** Effect of treating nuclear-donor cells with VPA on post-implantation developmental of clones.

Groups	Transferred embryos N	Pregnancies N (%)				Live born offspring
		Day 30	Day 60	Day 90	Day 270	Day 289
Control	34	7 (20.58)	3 (8.82)	3 (8.82)	3 (8.82)	3 (8.82)
VPA	30	6 (20.00)	2 (6.66)	2 (6.66)	2 (6.66)	1 (3.33)

Values within the same column did not differ statistically.

Pregnancy rate: number of pregnancies/number transferred embryos.

### Analysis of the health status of cloned calves

**Figure 5:**
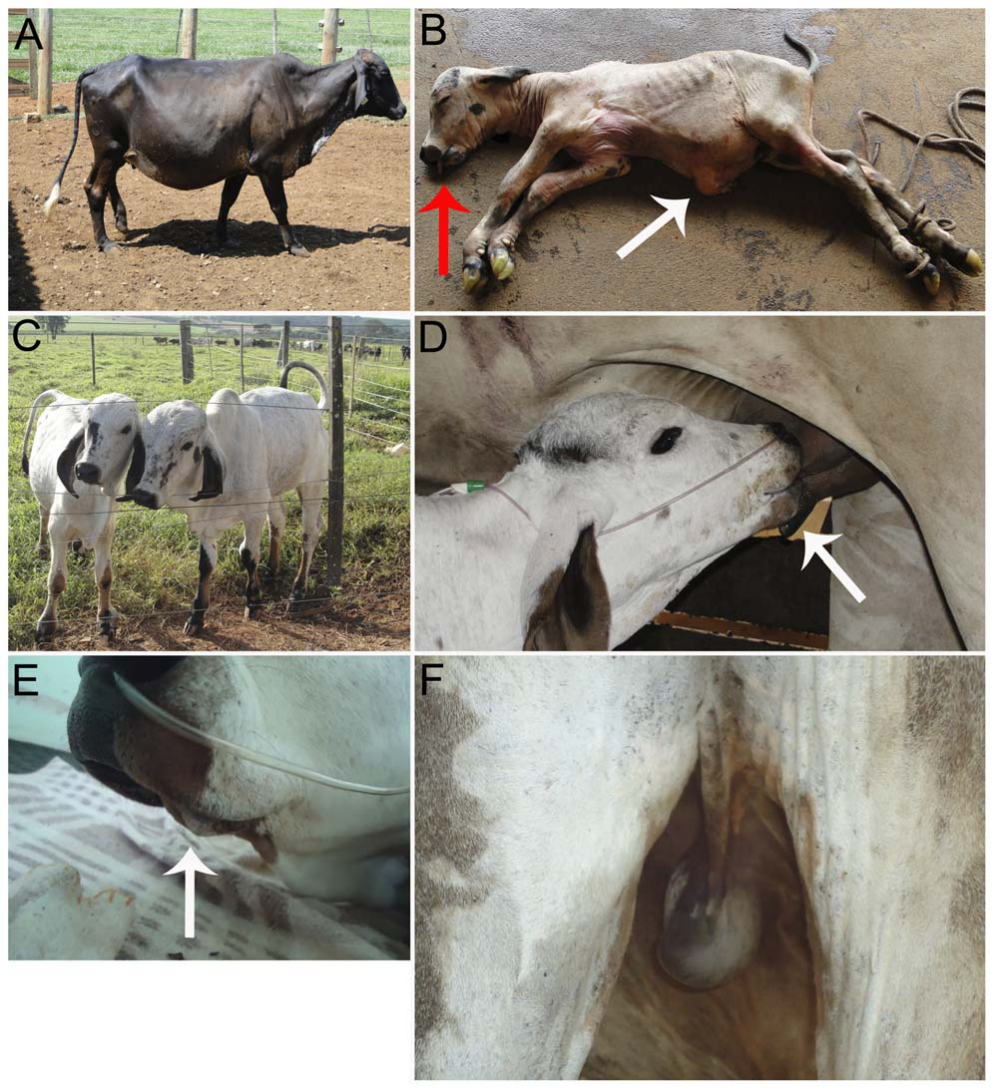
Analysis of health status of cloned calves. (A) Recipient female from the VPA-treated group showing signals of severe hydroallantois on day 270 of gestation. (B) Stillborn calf from VPA-treated group with enlarged umbilical cord and ascites (white arrow) and brachygnatism (red arrow). (C) Viable calves from the control group. (D) Calf from control group nursing, picture highlighting the correct morphology of the mandible (white arrow). (E) Mandible of cloned calf from control group with moderate brachygnatism (white arrow). (F) Picture highlighting the inguinal region of the calf from the control group evidencing monorchidism.

Five calves were born after SCNT; two from the VPA group and three from the control group (Table 3). The recipient that delivered one of the calves from the VPA group showed signs of severe hydrallantois on day 270 of gestation (Figure 5A). We performed an emergency cesarean section, and delivered a stillborn calf, in spite of intensive care management and attempts of resuscitation. The calf was born with enlarged umbilical cord and ascites (Figure 5B). The other four calves that developed to term were delivered by cesarean section as well, but on day 289 of pregnancy. Among these, the remaining calf from the VPA group presented intestinal atony and died 12 h after birth due to gastrointestinal complications. One calf from the control group, showed poor viability after birth, received intensive care, including intranasal oxygen supplementation and colostrum via an esophageal tube, but died seven days after birth from septicemia secondary to omphalitis. The other two calves from the control group had no apparent abnormality, showed strong suckling reflex and drank colostrum with vigor. Both had clinical parameters monitored (e.g. glycemia, respiratory and heart rate, rectal temperature, blood gas) during the first 24 h. After this period, as they showed no clinical abnormalities they were discharged to a ventilated and clean stall. At the time of writing this manuscript, the surviving clones (both from the control group) were 13 months old, healthy and normal (Figure 5C) compared to their age-matched peers derived from natural reproduction at the time of preparation of this manuscript. It is noteworthy that between the two survivors calves, one is morphologically normal, adjusted all vital parameters and nursed the surrogate cow (Figure 5D) faster than the other calf. Surprisingly, the four calves (two from VPA and two from control) had some degree of brachygnathism (Figure 5B and 5E). Furthermore, the surviving calf that had brachygnathism also had monorchidism (Figure 5F).

## Discussion

Almost two decades have passed since the first mammal was cloned by using donor cells from an adult animal [1]. Yet, SCNT is still an inefficient procedure in which less than 10% of the embryos transferred to recipients results in the birth of viable offspring [2], [42], [43]. Among the factors affecting SCNT efficiency, chromatin compaction is thought to be a challenge for reprogramming donor cells as it acts as an epigenetic barrier to complete nuclear reprogramming [9], [11]. HDAC inhibitors such as VPA are molecules that increase the global levels of histone acetylation [25] and the treatment of donor cells with HDAC inhibitors have been reported to effectively increase in vitro cloning efficiency [15], [19]. Thus, we hypothesized that the treatment of donor cells with VPA should increase the global levels of histone acetylation, leading to an “open chromatin” state. The use of these cells in SCNT might result in increased development rates of SCNT as their nuclei are expected to be more amenable to reprogramming [9], [25]. To address this hypothesis, we investigated the effects of donor cells treatment with VPA on cellular proliferation/viability, epigenetic remodeling, gene expression, and pre- and post-implantational development of cattle clones derived from them. Our results showed that, albeit VPA treatment increased cellular proliferation/viability, expression of *PPARGC1A* and *IGF2R*, and the levels of H3K9ac in donor cells, no effect on efficiency of SCNT was found when treated cells were used as nuclear donors.

The viability of the cells used as nucleus donors is a major point in SCNT as their nuclei drive development of SCNT embryos after oocyte reconstruction. Previous studies have shown that development of clones is negatively impacted when donor cells are treated with chromatin modifying agents (CMAs) that compromise cell cycle distribution, morphological characteristics and cellular proliferation [19], [29], [44], [45]. Therefore, donor fibroblasts were treated for 24 h with increasing concentrations of VPA (1, 2 and 5 mM) and evaluated based on an MTT assay. Since all doses resulted in significantly higher proliferation/viability according to the MTT assay, 2 mM of VPA was used in the subsequent experiments as this concentration has been shown to increase the rates of induced pluripotent stem cells (iPSCs) [21] production and SCNT in mice [14]. It is noteworthy that based on the MTT assay, the treatment with VPA increased cell proliferation/viability while these cells were not expected to proliferate because of serum starvation to synchronize the cell cycle [46], [47]. However, this increase might be caused as a side effect of VPA on cell metabolism as the MTT assay is based on activity of metabolic enzymes that reduces tetrazolium salts [30]. According to a recent paper, inhibition of HDAC doubled the activity of enzymes related to intermediate cell metabolism [48]. *PPARGC1A* is a transcriptional coactivator with a central role in mitochondrial biogenesis and metabolism regulation in the cell [41]. The finding that VPA treatment increased expression of *PPARGC1A* further supports its effect on cell metabolism as previously reported [49], [50]. Based on these findings, 2 mM of VPA were found to be a suitable concentration to be used in the subsequent experiments.

In a second experiment we confirmed the effect of VPA on histone acetylation by evaluation of fibroblasts treated with 2 mM of VPA for 24 h in comparison to untreated cells. In addition, since VPA has been shown to cause DNA demethylation [38], the global levels of DNA methylation were evaluated, but no difference was found between control and treated cells. Since VPA causes demethylation by stimulating accessibility of a demethylase to DNA [38], we believe that a more prolonged treatment may be needed in order to obtain detectable levels of DNA demethylation. Next, since we hypothesized that the effects of VPA treatment would persist into embryo development, we attempted to confirm that the higher acetylation levels of donor cells were maintained after nuclear transfer. However, similar levels of histone acetylation were found between cloned embryos produced with control and treated cells. Moreover, a significant decrease in acetylation levels was found in SCNT couplets at 5 h.p.a. when fibroblasts treated with VPA were used as nuclear donors. This is in contrast with a report in the mouse, in which histone modifications caused by treatment of 8-cell embryos with VPA were maintained at least until the blastocyst stage [51]. On the other hand, oocytes are known to have special enzymatic activities, such as histone-modifying and DNA demethylating enzymes [11], that are responsible for reprogramming the sperm after fertilization [52]. Gao et al. showed that reprogramming by nuclear transfer uses the developmental program that is normally used after fertilization, termed by the authors as “erase-and-rebuild” process [53]. Furthermore, we speculate that the oocyte might have erased the epigenetic marks brought by the transferred nucleus, to reestablish a new developmental program. According to our data, this erasing process begins immediately after nuclear transfer, as no difference of acetylation was seen between control and treated groups immediately before artificial activation (approximately 1 to 2 h after couplet fusion). The ability of the oocyte to reverse the hyperacetylation caused by VPA, lowering the acetylation levels compared to a control group (5 h.p.a.), raises questions about the relevance of pretreating donor cells with CMAs. Enright et al. also found hypoacetylation in SCNT embryos produced by treatment of donor cells with TSA [19]. The authors suggested this was caused by a rebound effect from drug treatment, because the effect of HDACis is reversible [19]. In rabbits, similar results were found after treatment with NaBu and the authors argued that the aberrant epigenetic marks of clones cannot be corrected by the treatment with HDAC inhibitors [18]. In summary, although the treatment of donor cells with VPA caused histone hyperacetylation, this epigenetic state was reversed after oocyte reconstruction resulting in zygotes from treated group with lower levels of H3K9ac than the control group.

In a third experiment we investigated the effect of VPA treatment on gene expression in fibroblasts. It was reported that the treatment with VPA, even for a short period, had a significant effect on gene expression in cultured cells [25], [54]. Since treatment of donor cells has been reported to improve SCNT rates [15], [28], we hypothesized that this effect might be mediated, among other factors, by an effect of VPA on expression of key genes to SCNT. Since an appropriate expression of *POU5F1* [55] and *IGF2R* [56] during early embryogenesis is critical to the success of SCNT, we evaluated whether the expression of these genes is altered in fibroblasts treated with VPA. We found no effect of the treatment on the expression of *POU5F1*, but VPA did increase expression of *IGF2R* compared to untreated cells. *POU5F1* plays an essential role in controlling cellular pluripotency and therefore is a key factor in nuclear reprogramming in SCNT [40]. Treatment of myogenic cells with VPA was been shown to increase expression of *POU5F1* [57]. Thus, SCNT might benefit from an overexpression of *POU5F1* in donor cells as SCNT embryos frequently fail to express this gene [55]. However, as we found that VPA did not affect *POU5F1* expression, we believe that other epigenetic modifications than histone acetylation are involved in the control of *POU5F1* expression [58]. With respect to *IGF2R*, this gene plays an important role as a negative effector in fetal growth since it promotes *IGF2* arrest into lysosomes followed by degradation [59], [60]. It has been hypothesized that dysregulation of imprinted genes such as *IGF2R* are the cause of poor developmental rates observed in SCNT [56], and alterations of *IGF2R* expression has often been associated to common problems in cloned embryos [61]. For instance, in sheep, parthenogenetic embryos that express low levels of *IGF2* and high levels of *IGF2R* and *H19*, have retarded growth when compared to control embryos [62]–[64]. On the other hand, the reduced *IGF2R* expression in ovine embryos cultured in vitro is associated with Large Offspring Syndrome [39]. Baqir et al. observed that most imprinted genes have their expression increased following exposure of mouse embryonic stem cells to TSA [65]. Interestingly, they also found that expression of several imprinted genes remained high and in some cases, increased further, after drug removal or even after cell passageing, indicating a long lasting and retarded effect of the treatment on gene expression [65]. Herein we confirmed the effect of VPA on increased expression of an imprinted gene, providing further evidence that acetylation is involved in regulation of imprinting [65], [66]. The higher expression levels of *IGF2R* induced by the treatment of donor cells with VPA might affect SCNT as the epigenetic modifications induced by VPA on *IGF2R* have a long lasting effect on development [65]. Hence, the treatment of donor cells with VPA resulted on increased expression of *IGF2R* without affecting expression of *POU5F1*.

Although the hyperacetylation caused by VPA on donor cells was reversed after nuclear transfer, we decided to evaluate development of cloned embryos to further characterize the effect of VPA on SCNT. It is possible that some of the modifications induced by VPA on donor cells (e.g. overexpression of *IGF2R*) remain after nuclear transfer, with a consequent effect on cloning efficiency. Several groups have reported increased rates of SCNT when donor cells were treated with HDAC inhibitors, including VPA [28], TSA (cattle) [19], [32] and NaBu (cattle [15] and rabbits [18]). Yet, here we found no effect of donor cells treated with VPA on pre-implantation development of SCNT embryos. Although surprisingly this finding is in agreement with a previous reports in which NaBu was used to produce pig clones [45]. This report described that the treatment of donor cells with NaBu resulted in histone hyperacetylation, but the use of these cells in SCNT did not affect development of cloned embryos. These contradictory results provide evidence that the effect of CMAs may diverge depending on several factors including species and reprogramming system (e.g. SCNT or iPSCs). This notion is supported by a recent report with cloned mice which showed that several HDAC inhibitors such as TSA, scriptaid, suberoylanilide hydroxamic acid and oxamflatin reduced the rate of apoptosis in blastocysts and improved full term development, whereas VPA had no effect on SCNT efficiency [67]. The authors argued that VPA is an inhibitor of HDAC classes I and IIa, whereas the others are inhibitors of HDAC classes I and IIa/b, suggesting that inhibition of HDAC class IIb is a key step for successful reprogramming in the mouse [67]. In contrast, Costa-Borges et al. found that VPA improved in vitro development, blastocyst quality, and full term development of cloned mice, at comparable level to TSA [14]. Treatment with VPA also improved pre-implantation development of cattle [68] and pigs [69] when the cloned embryos were treated during in vitro culture. The treatment resulted in an increase of blastocyst rate and cell number in the inner cell mass (ICM) [68], [69]. Selokar et al showed that the treatment of donor cells with VPA improved bovine SCNT blastocysts production, reduced the apoptosis and H3K9 methylation levels, similar to those of embryos derived from IVF [28]. While these results differ from our, it should be noted that Selokar et al used handmade cloning, which is characterized by significant differences in the technique from that used in the present study, which might explain the differences observed in pre-implantation development. In addition, VPA was recently found to regulate pluripotency in iPSCs, increasing the efficiency as measured by the number of colonies, and up-regulation of pluripotency genes [21]. In summary, the effect of CMAs such as VPA on SCNT is not clear [70], but here we found no effect of VPA on developmental rates of SCNT embryos.

Taking into account that VPA might have affected SCNT embryos later during development, the blastocysts derived from the previous experiment were transferred to recipients to evaluate post-implantation development. Cloning of cattle by SCNT is typically associated with a high incidence of pregnancy failure throughout gestation [7], [71]. Our data are in accordance with previous reports, showing that the majority of established pregnancies (60–70%) were lost around the time of implantation [56], [72]. In cloned cattle, the losses that occur between days 30 and 60 of pregnancy are frequently associated to morphological (placentomegaly and faulty vascularization) and functional (steroidogenesis) abnormalities in placentas [71]. With respect to VPA, no effect of its treatment was seen on post-implantation development as the rate of pregnancy failure between 60 and 270 days and rate of development to term did not appreciably differ between groups. This unexpected result supports the hypothesis that all the modifications induced by the treatment were either inconsequential for post blastocyst development or reversed after nuclear transfer. Moreover, whereas two out of three clones produced with untreated cells were healthy at birth, the two clones that derived from VPA-treated cells died after caesarian section. This highlights the importance of evaluating post-implantation development and survival rate when CMAs are employed in SCNT. This agrees with the report by Kang et al. with pigs who described that VPA improved in vitro development of cloned embryos but did not improve survival to adulthood [73]. Interestingly, herein four out of five calves produced by SCNT (two from untreated cells and two from treated cells) presented brachygnatism. This trait is widely accepted to be a congenital and heritable abnormality [74]. However, the bull whose donor cells were derived did not present this phenotype. We speculate that the cell culture environment or an incomplete nuclear reprograming might have led to such abnormality. In a previous study, Johnson et al. also reported the occurrence of brachygnatism in foals derived by SCNT, trait that was not present in the horse used to donor cell derivation [74]. Strikingly, one of the viable calves, in addition to brachygnatism, had monorchidism whereas the other calf did not present noticeable abnormalities. After birth, the calf without abnormalities, stood up, drank colostrum and adjusted the physiological parameters faster than the other one with brachygnatism and monorchidism. This suggests that the process of nuclear reprogramming is stochastic, with calves derived from the same cell line presenting variable levels of “reprogramming” and health status. In summary, the treatment of donor cells with VPA did not affect developmental rates resulting in one stillborn and one calf that survived only 12 h after birth.

In conclusion, accumulated evidence suggests that CMAs are highly important to aid nuclear reprogramming with a consequent increase in SCNT efficiency in the mouse [75]. In cattle the use of CMAs remains largely controversial [70], [76]. Altogether, our results show that in spite of the alterations caused in fibroblasts by the treatment with VPA, their use as donor cells in SCNT does not improve pre- and post-implantation development of cloned cattle. In the future, it will be interesting to dissect the roles that CMAs have in donor cells and cloned embryos, to gain mechanistic insights on their use.
